# Microbiota of the Gut-Lymph Node Axis: Depletion of Mucosa-Associated Segmented Filamentous Bacteria and Enrichment of *Methanobrevibacter* by Colistin Sulfate and Linco-Spectin in Pigs

**DOI:** 10.3389/fmicb.2019.00599

**Published:** 2019-04-11

**Authors:** Benjamin Zwirzitz, Beate Pinior, Barbara Metzler-Zebeli, Monika Handler, Kristina Gense, Christian Knecht, Andrea Ladinig, Monika Dzieciol, Stefanie U. Wetzels, Martin Wagner, Stephan Schmitz-Esser, Evelyne Mann

**Affiliations:** ^1^Institute of Milk Hygiene, University of Veterinary Medicine, Vienna, Austria; ^2^Austrian Competence Centre for Feed and Food Quality, Safety and Innovation, FFoQSI GmbH, Tulln an der Donau, Austria; ^3^Institute for Veterinary Public Health, University of Veterinary Medicine, Vienna, Austria; ^4^University Clinic for Swine, University of Veterinary Medicine, Vienna, Austria; ^5^Institute of Animal Nutrition and Functional Plant Compounds, University of Veterinary Medicine, Vienna, Austria; ^6^Department of Animal Science, Iowa State University, Ames, IA, United States

**Keywords:** antibiotics, lymph nodes, ileum, microbiome, 16S rRNA gene, metatranscriptome, segmented filamentous bacteria, gut microbiota

## Abstract

Microorganisms are translocated from the gut to lymphatic tissues via immune cells, thereby challenging and training the mammalian immune system. Antibiotics alter the gut microbiome and consecutively might also affect the corresponding translocation processes, resulting in an imbalanced state between the intestinal microbiota and the host. Hence, understanding the variant effects of antibiotics on the microbiome of gut-associated tissues is of vital importance for maintaining metabolic homeostasis and animal health. In the present study, we analyzed the microbiome of (i) pig feces, ileum, and ileocecal lymph nodes under the influence of antibiotics (Linco-Spectin and Colistin sulfate) using 16S rRNA gene sequencing for high-resolution community profiling and (ii) ileocecal lymph nodes in more detail with two additional methodological approaches, i.e., cultivation of ileocecal lymph node samples and (iii) metatranscriptome sequencing of a single lymph node sample. Supplementation of medicated feed showed a local effect on feces and ileal mucosa-associated microbiomes. Pigs that received antibiotics harbored significantly reduced amounts of segmented filamentous bacteria (SFB) along the ileal mucosa (*p* = 0.048; 199.17-fold change) and increased amounts of *Methanobrevibacter*, a methanogenic Euryarchaeote in fecal samples (*p* = 0.005; 20.17-fold change) compared to the control group. Analysis of the porcine ileocecal lymph node microbiome exposed large differences between the viable and the dead fraction of microorganisms and the microbiome was altered to a lesser extent by antibiotics compared with feces and ileum. The core microbiome of lymph nodes was constituted mainly of *Proteobacteria*. RNA-sequencing of a single lymph node sample unveiled transcripts responsible for amino acid and carbohydrate metabolism as well as protein turnover, DNA replication and signal transduction. The study presented here is the first comparative study of microbial communities in feces, ileum, and its associated ileocecal lymph nodes. In each analyzed site, we identified specific phylotypes susceptible to antibiotic treatment that can have profound impacts on the host physiological and immunological state, or even on global biogeochemical cycles. Our results indicate that pathogenic bacteria, e.g., enteropathogenic *Escherichia coli*, could escape antibiotic treatment by translocating to lymph nodes. In general ileocecal lymph nodes harbor a more diverse and active community of microorganisms than previously assumed.

## Introduction

The gut-lymph node axis plays a key role in the symbiotic relationship between intestinal microbiota and the host immune system (Sansonetti, 2004; Kamada and Núñez, 2013). Despite the large number of microorganisms in the gastrointestinal (GI) tract, most of them do not induce harmful immune responses. This is particularly essential, because the mucus-associated microbiota sets strong stimuli on the intestinal epithelial barrier along with the underlying lamina propria. Gut-derived dendritic cells (DCs) sample commensal microbes either directly from the GI mucus layer, which represents the interface between the lumen and enterocytes, or via specialized microfold cells (Macpherson and Uhr, 2004; Pickard and Chervonsky, 2010; Macpherson et al., 2012; Ohno, 2016). Subsequently, DCs transport microorganisms to mesenteric lymph nodes – the key immune inductive site for microbial translocation control (Macpherson and Uhr, 2004; Macpherson and Smith, 2006). In contrast to macrophages that immediately kill most bacteria, DCs hold living bacteria and are known to be ineffective in eliminating them, thus preserving antigenic information for the immune system (Nagl et al., 2002). It is assumed that commensal bacteria can survive within DCs migrated to mesenteric lymph nodes for several days or weeks (Nagl et al., 2002; Macpherson and Uhr, 2004; Hooper et al., 2012; Gorvel et al., 2014). Our previous findings based on cultivation and cDNA amplicon sequencing of lymphatic tissues provide evidence that lymphatic tissues harbor a high diversity of living bacteria (Mann et al., 2014a, 2015a), which was also shown for lymph nodes of healthy mule deer (Wittekindt et al., 2010).

Under conditions of antibiotic-induced dysbiosis, translocation processes of bacteria to mesenteric lymph nodes are supposed to be altered, including non-invasive bacteria that were additionally trafficked by mononuclear phagocytes (Diehl et al., 2013). To improve feed efficiency or to prevent and treat diseases, antibiotics are widely used in animal husbandry. Since the European Parliament’s Environment and Public Health Committee has decided to support a ban on prophylactic and growth promoting antibiotics used in pig production for prevention of diseases and promotion of growth (MEPs vote to ban prophylactic use of antibiotics in animals, 2016), in-feed antibiotics have been under constant scrutiny (Diana et al., 2017). Primary concerns are resistance genes in microbiomes, which might develop, disseminate and persist in antibiotic-fed animals and their environment (Looft and Allen, 2012). In weaner pigs, in-feed antibiotics were shown to have only minimal effects on pig health and welfare (Diana et al., 2017). In adult pigs, the in-feed antibiotic ASP250, but not carbadox, caused significant microbiota shifts in the feces, such as increasing the abundance of *Escherichia (E.) coli* (Looft et al., 2012). Recent findings suggest that the in-feed antibiotics monensin and tylosin reduce the abundance of some Gram-positive genera but do not induce changes at phylum level in pigs as well as in cattle (Kim et al., 2012; Thomas et al., 2017).

This study aimed to characterize shifts in microbial community structure across the intestinal mucosa-enteric lymph node axis in order to understand the effects of two commonly used antibiotics (Linco-Spectin and Colistin sulfate; oral application). We hypothesized that these antibiotics influence GI microbiota diversity and community structure and that, as a result, the ICLN-ileum axis is altered through permeability changes and changed translocation patterns. Medicated-feed antibiotics are still heavily used, not only in the E.U. but worldwide in case of infections. In addition to the taxonomic community profiling survey with 16S rRNA gene amplicons, we sequenced the metatranscriptome of a single ICLN sample to reveal first insights into the functional aspects of the bacterial microbiome in ICLNs. Our analyses therefore fill a relevant knowledge gap with regard to the relationship between the GI tract- and the ICLN-associated bacterial microbiome.

## Materials and Methods

### Ethics Statement

Animal handling and treatment were discussed and approved by the institutional ethics and animal welfare committee (ETK-03/04/2015; University of Veterinary Medicine, Vienna, Austria) in accordance with GSP guidelines and national legislation (paragraph 8ff of Law for Animal Experiments, Tierversuchsgesetz).

### Animals and Experimental Design

Sixteen female pigs [(Large White × Landrace) × Piétrain] aged 6 months were used in this experiment. Prior to the experiment, pigs were housed together in a pen for 3 months and were fed with a commercial corn-soybean meal diet for growing pigs (Garant Qualitaetsfutter, Pöechlarn, Austria) (Supplementary Table S1). Pigs were housed on straw and had free access to demineralised water. The pen was separated into two equally sized boxes using stainless steel separators. Pigs were randomly allocated to two groups (*n* = 8 per group). One group, further named as antibiotic-treated (AB) group, received the diet with medicated feed (Linco-Spectin and Colistin Enteromix) and the other one served as a control group receiving the diet without antibiotics. The feed was not sterilized but all pigs were fed from the same feed lot to standardize for potential effects of the natural feed microbiome on the intestinal microbiome.

### Feed Preparation and Antibiotic Dosages

Feed was prepared based on an average pig weight of 100 kg and a daily feed intake of 2.50 kg per pig. Antibiotics were premixed with 20 kg standard corn-soybean meal diet in a mixing machine (Betonmischer, Lagerhaus, Vienna, Austria). The recommended and used daily dosage for the agent Colistin sulfate (AniMed Service, Dobl, Austria) was 5 mg/kg body weight, for Linco-Spectin (Pfizer, Vienna, Austria) it was 125 mg agent premix/kg body weight. Therefore, 160 g Colistin Enteromix and 100 g Linco-Spectin premix were mixed with 20 kg feed. Pigs were fed twice a day at 7 a.m. and 5 p.m. Each pig group received 20 kg feed per day and experimental diets were fed until pigs were sampled.

### Sampling of Porcine Feces, Ileal Scrapings, and Ileocaecal Lymph Nodes

Fecal samples were taken on the evening before the experiment started (sample name: feces – start) and on day 22 (sample name: feces – end) and stored at -80°C. From day 22 to day 25, four pigs were sacrificed and sampled per day, including two animals from each experimental group. Pigs were killed by captive bolt stunning in accordance with the Austrian National Authority guideline. The arteria carotica and the arteria subclavia were opened immediately to ensure complete exsanguination. Afterward, the abdomen was cut open, ileocecal lymph nodes were dissected bluntly and fat and connective tissue was removed. Two ICLNs were transferred into a 50 ml polypropylene tube (Corning, Corning, NY, United States), and stored in liquid nitrogen (sample name: ICLN), the other ICLNs were transferred into sterile polypropylene tubes and stored on ice for cultivation experiments. Subsequently, the ileum was identified and removed. The distal 10 cm of the ileum were opened and washed a few times in sterile ice-cold 1 × phosphate buffered saline (Merck, Darmstadt, Germany) until it was cleaned from digesta, before scraping off the mucosa with a microscopic glass slide, which was placed into tubes, and stored in liquid nitrogen (sample name: ileum). For the measurement of small intestine electrophysiological parameters and permeability marker flux rates, a jejunal tube piece (20 cm) was taken proximal to the ileal tissue sample taken for the microbiota analysis. This tissue piece was immediately transferred into ice-cold transport buffer (Metzler-Zebeli et al., 2017) which was pre-gassed with carbogen (95% O_2_ and 5% CO_2_). The procedure from pig stunning until the end of sampling took between 15 and 20 min per pig.

### Cultivation of ICLN Samples and Processing of Isolates

On the day of the euthanasia ICLNs stored on ice were cultivated immediately or not later than 3 h after sampling. ICLNs were plunged into 96.00% ethanol and flamed shortly. One ICLN of each pig was cut medially with a sterile scalpel blade and a 0.25–0.50 g piece was cut out from the middle of the ICLNs. The piece was transferred into a plastic bag with 10 ml 1 × sterile PBS, pH 7.2 and was homogenized with a Stomacher 400 blender (Steward, London, United Kingdom). For the cultivation, tryptone soya-yeast agar (TSA-Y; Noack & Co AG, Vienna, Austria), a minimal medium made with 1/10 TSA-Y Agar (Noack & Co AG, Vienna, Austria), Columbia agar with 5.00% sheep blood (COL; BioMerieux, Vienna, Austria), and self-prepared ICLN agar (Supplementary Table S2) was used. One hundred microliters of each homogenate were plated and then cultivated both aerobically and anaerobically at 37°C for 2 days. Anaerobic incubations were done in anaerobic chambers containing two GENbox Anaer bags (bioMérieux, Nürtingen, Germany). After 2 days, all well-separated single colonies were picked and re-cultivated on a new plate of the same type and incubated again at 37°C. Bacterial isolates were stored at -80°C in glycerol (20.00% vol/vol). DNA isolation of the cultures was performed using the NucleoSpin Extract Kit^®^ (NucleoSpin Extract Kit; Macherey-Nagel, Düeren, Germany) as described by the manufacturer. A standard 16S rRNA gene PCR (initial denaturation at 95°C for 5 min, then denaturation at 94°C for 40 s, annealing at 52°C for 40 s and elongation at 72°C for 1 min, 35 cycles, final elongation at 72°C for 7 min) was used with the primers 27F (5′-AGAGTTTGATCMTGGCTCAG-3′, positions 8 to 27 in *E. coli* 16S rRNA coordinates) and 1492R (5′-GGY TAC CTT GTT ACGACT T-3′, positions 1492 to 1510 in *E. coli* 16S rRNA coordinates) (Weisburg et al., 1991). PCR products were purified (Gene JET PCR Purification Kit, Thermo Fisher Scientific GmbH, Vienna, Austria) and Sanger sequenced from both directions (LGC Genomics, Berlin, Germany; Microsynth, Austria). In total, 95 isolates had a high-quality chromatogram and were included in the analysis presented here. Sequences were blasted against the NCBI GenBank excluding uncultured and environmental sample sequences in the search set to define a closest reference strain. Furthermore, isolate sequences were compared with the Illumina 16S rRNA gene sequencing representative OTUs. Sequences from one representative sequence from each Illumina OTU (rep_set) were searched against a database containing near full-length 16S rRNA gene sequences obtained from the cultivation approach using BLASTn (Reference Blast). Complementary, all isolates affiliated to *E. coli* were screened for virulence genes (*fli*C, *stx*1, *stx*2, *eae, rfb*E, and *hly*A) with a multiplex PCR according to Bai et al. (2010).

### Gut Electrophysiology

The electrophysiology of the small intestine was performed as previously described (Metzler-Zebeli et al., 2017). Three successional samples from a jejunal tube piece were evaluated in parallel. After opening each tissue piece at the mesenterium, it was rinsed with transport buffer to remove remaining digesta particles, stripped of the outer serosal layers (tunica serosa and tunica muscularis) and mounted in one Ussing chamber. The time elapsing until the tissue pieces were mounted into the Ussing chambers was between 15 and 30 min. Briefly, each replicate tissue piece (0.91 cm^2^) was bathed in experimental buffer solution (38°C) and gassed with carbogen [95% O_2_ to 5% CO_2_; (Metzler-Zebeli et al., 2017)]. Each Ussing chamber was connected to a pair of dual channel current and voltage electrodes (Ag–AgCl) which were submerged in 3% agar bridges filled with 3 M potassium chloride. The tissue was alternatively pulsed with a positive or negative pulse of 20 μA for 100 ms of duration. The tissues were allowed an equilibration period of 20 min under open-circuit conditions after which the tissue was short-circuited by clamping the voltage to zero. The potential difference (mV), short-circuit current (I_Sc_, μA/cm^2^) and transepithelial resistance (Ω × cm^2^) were continuously recorded using a microprocessor-based voltage-clamp device and software (version 9.10; Mussler, Microclamp, Aachen, Germany). The tissue conductance (G_T_, mS/cm^2^) was calculated as the reciprocal of the R_T_.

The electrophysiological measurements were recorded for 5 min, then fluorescein 5(6)-isothiocyanate (FITC, final concentration: 0.1 mmol/L; Sigma-Aldrich, Schnelldorf, Austria) and horse-radish peroxidase (HRP, final concentration: 1.8 μmol/L; Carl Roth GmbH + Co.KG, Karlsruhe, Germany) were added to assess the mucosal-to-serosal flux of the gut as described in Metzler-Zebeli et al. (2017). After 185 min after the voltage clamp, theophylline (inhibitor of the phosphodiesterase; final concentration, 8 mM) was added to both chamber sides to monitor tissue vitality. Normality was established using the Shapiro–Wilk test in SAS (version 9.4; SAS Inst. Inc., Cary, NC, United States). Thereafter, the electrophysiology data were analyzed by ANOVA using the MIXED procedure in SAS. Fixed effects included in the model were group (control vs. AB group). The pig was the experimental unit. Degrees of freedom were approximated by the method of Kenward-Roger. The Tukey correction for multiple testing was used for pairwise comparisons between least squares means. Least squares means were computed and significance declared at *p* < 0.05.

### Sample Preparation, DNA Extraction, 16S rRNA Gene Sequencing, and Bioinformatics

The 16S rRNA gene sequencing was performed with 16 fecal samples taken prior the start of the experiment (feces-start), 16 fecal samples taken at day 22 before sacrificing started (feces-end), 16 ileocecal lymph node samples (ICLN), 16 ileum scraping samples (ileum), and one extraction control sample (NTC, no template control).

Prior to DNA extraction, a sample preparation method initially invented for food matrices was applied for ICLNs and ileum samples to remove inhibitors and to enrich bacterial cells out of a large amount (1.2 g) of tissue (Mayrl et al., 2009; Mann et al., 2014b). The sample preparation procedure was controlled with 16S rRNA gene-targeted PCRs of negative controls (one negative control per batch). For each sample, 1.2 g of tissue (ileum or ICLN) was homogenized for 10 min with a Stomacher 400 blender (Steward, London, United Kingdom) in 35 ml 2M MgCl_2_, 50 mM Tricine pH 7.60, and 1.00% Lutensol AO-07 (Mester et al., 2014) and processed for every sample according to the protocol. After the first incubation step at 37°C, an additional filtering step (mesh diameter 1 mm) was applied for ICLNs to remove free connective tissue, which is not solvable in the lysis buffer. For the DNA extraction, the pellets were subsequently incubated with 180 μl lysis buffer (20 mM Tris/HCl; 2 mM EDTA; 1% Triton X-100; pH: 8) supplemented with lysozyme (20 mg/ml) (Sigma-Aldrich, Vienna, Austria) with constant shaking for 1 h at 37°C and incubated again with Proteinase K (25 μl, 20 mg/ml) over night at 56°C. For processing fecal samples, 200 mg feces were mixed with 1 ml 1 × PBS. Undigested plant particles were removed by a 30 s centrifugation step at 3000 × *g*. The supernatant was then centrifuged for 8 min at 20,000 × *g* and for the DNA extraction, the pellet was subsequently incubated with lysis buffer (same as for ICLNs) for 1 h at 37°C and incubated with Proteinase K (25 μl, 20 mg/ml) over night at 56°C. The DNA of all samples was extracted with the NucleoSpin^®^ tissue kit (Macherey-Nagel, Düeren, Germany) according to the manufacturer’s instructions.

For amplicon sequencing, the 16S rRNA gene hypervariable region V4 was targeted using an Illumina MiSeq sequencing platform (Microsynth AG, Balgach, Switzerland) with a 250 bp paired-end read protocol. The run was internally controlled by Microsynth by sequencing a mock community.

Library preparation, including sample quality control and Nextera two-step PCR amplification with the primer set 515F/806R (Kroes et al., 1999; Paster et al., 2001), equimolar pooling of samples, and sequencing were performed by Microsynth. Sequence data were analyzed with the software package QIIME v1.9 (Caporaso et al., 2011). ICLN samples were trimmed to 242 bp, due to decreasing quality scores at the end of the sequences.

Sequences with a Phred score <19 were filtered and chimeric sequences were excluded (0.06%) by using the USEARCH 6.1 database (Edgar, 2010). Fecal and ileum samples were sequenced deeper compared with ICLN samples (Supplementary Figure S1: For fecal and ileum samples, 335 868 sequences/sample were generated (median value), whereas for ICLNs, 12 241 sequences/sample were generated (median value). This difference is due to the lower absolute number of bacteria in ICLNs compared with mucosa or gut content and due to many PCR inhibitors, which we mostly got rid of with our extensive pre-extraction protocol (Cardoso et al., 2009). The challenge of detecting rare microbiota in the dominant pool of host genetic material in lymph nodes was described before (Wittekindt et al., 2010). Finally, sequences were clustered into operational taxonomic units (OTUs; 97% similarity) with the QIIME script “pick_open_reference_otus.py.” Taxonomy of OTU representative sequences was assigned with the Greengenes database (version 13_5). Additionally, the 50 most abundant OTUs were also aligned against the refseq_rna database excluding all uncultured environmental sequences, which revealed multiple species with the same sequence identity and similarity score for most of these OTUs. Therefore, we affiliated them with the closest secured taxonomy rank. OTUs with less than 10 sequences were removed, resulting in 5,394 OTUs. Contaminant OTUs were detected with the R package “decontam” using a prevalence-based contaminant identification with a *p*-value cutoff of 0.1 (Davis et al., 2018). We constructed an OTU network using the make_OTU_network.py script in QIIME and a yFiles-organic layout in Cytoscape (Shannon et al., 2003). VENN diagrams were calculated with shared_phylotypes.py in QIIME. Relative abundance plots and rarefaction curves of microbial communities were calculated within the phyloseq package and visualized with ggplot2 in R (McMurdie and Holmes, 2013; Wickham, 2016). Weighted Unifrac analysis was done with a rarefied dataset including 4,080 sequences per sample (smallest library size among all samples) and was based on the calculation of pairwise sample dissimilarity using the QIIME script “beta_diversity.py.” Normal distribution of data and residues was tested with the Shapiro–Wilk normality test in R. To determine significant difference in means between AB and Control group, a one-way ANOVA for normal distributed and a Kruskal–Wallis test for not normal distributed data, with subsequent Benjamini-Hochberg False Discovery Rate correction (FDR), was performed.

### Biostatistical Analysis of the Microbiota Composition

Biostatistical analysis was implemented using the R statistical computing environment (R version 4.3.3, 2017). Alpha diversity indices were assessed by calculating the Chao1 index, Shannon diversity index, Simpson’s index of diversity, and the OTU richness of the full dataset. Due to uneven library sizes, the dataset was rarefied at the depth of the smallest library (4,080 sequences) for statistical analysis of diversity indices. We used lme models (linear mixed-effect models, R-package, nlme (Pinheiro et al., 2017), to analyze the effects of the independent variables, i.e., sampling sites (feces-start, feces-end, ileum, and ICLN) and group (AB or control) and their interaction on the abundance of diversity indices, considered as dependent variable, whereas the individual pigs were taken into account as random factor. The dependent variables were box-cox transformed. The residuals of the models were assessed visually as histograms, Q-Q-plots and by calculation of the Shapiro–Wilk normality test.

Further, the normality distributions of the abundance of phyla (*n* = 25), families (*n* = 145), and OTUs (*n* = 5,394) were checked with the tests for multivariate data in semi-parametric factorial designs with the function MANOVA.wide and corresponding residual plots (Friedrich et al., 2017). Due to not normal distribution of these data and residues, the beta diversity of phyla, families and OTUs was assessed by applying a permutational multivariate analysis of variance (PERMANOVA, formerly nonparametric MANOVA) with the adonis function and 5,000 permutations in R (Oksanen et al., 2019). To analyze whether independent variables had a significant effect on the composition of phyla, families and OTUs, we calculated the Bray-Curtis-dissimilarity matrix with the R function vegdist of the vegan package. The distance matrix was further applied as response variable. The multivariate homogeneity of group dispersions was performed with the betadisper function, followed by a permutation-based test of multivariate homogeneity of group dispersions with a pairwise comparison of group mean dispersions. Subsequently, the Kruskal–Wallis test was used to identify statistically significant difference between each individual phylum, family, and OTU abundance and independent variables and their interaction, followed by a pairwise test for multiple comparison of mean rank sum (Dunn’s test), adjusted with Benjamini-Hochberg method (Pohlert, 2014). Data are considered significant at *p* ≤ 0.05.

The weight of pigs in the AB group and control group were compared using the Wilcoxon rank sum tests with continuity correction for unpaired samples. The comparison of weight during the time of the experiment was tested with the Wilcoxon signed-rank test.

### Metatranscriptome Sequencing of an ICLN Sample

To test the bacterial transcriptional activity in lymph nodes, the RNA of a single lymph node sample (taken from pig no. 1 belonging to the control group) was subjected to standard Illumina library preparation using the NEBNext^®^ Ultra^TM^ RNA Library Prep Kit. rRNA was depleted with the Ribo-Zero^TM^ Magnetic Gold Kit (Epicentre Biotechnologies, Madison, WI, United States). The library was sequenced with a 50 bp paired-end protocol using an Illumina HiSeq2500 at the Vienna Biocenter Core Facilities VBCF NGS Unit (The Vienna Biocenter Core Facilities/Next Generation Sequencing). Sequences were quality filtered with mothur (Schloss et al., 2009) using trimming parameters as follows: number of ambiguous bases allowed = 0, minimum length of reads = 30, minimum average quality score allowed over a window = 25, bases in a window = 10, maximum of homopolymers = 8. RNA sequence analysis of the bacterial lymph node metatranscriptome was done with the Meta Genome Rapid Annotation using Subsystem Technology v4.0 server of the Argonne National Laboratories (Meyer et al., 2008; Keegan et al., 2016). Artificial duplicate reads were removed (Cox et al., 2010). Host reads mapped to the genome of *Sus scrofa* and rRNA sequences were filtered. Putative protein coding features were predicted using FragGeneScan (Rho et al., 2010). For downstream analysis, the Kyoto Encyclopedia for Genes and Genomes (KEGG) (Kanehisa and Goto, 2000; Kanehisa et al., 2016) and KEGG orthology (KO) systems were used with an *e*-value cutoff of 1 × 10^-5^, identity cut-off value of 60% and a minimum alignment length of 15.

## Results

### Growth Performance

Growth performance of pigs is listed in Supplementary Table S3. The average daily weight gain was 0.29 kg/day, with a significant increase in body weight during the experiment (*p* < 0.001). Pigs fed with the standard corn-based diets only (control group), did not differ significantly in body weight gain compared with antibiotic fed pigs (*p* = 0.833).

### Electrophysiological Measurement in the Small Intestine

Electrophysiological parameters (i.e., I_Sc_ and G_T_) of the duodenal mucosa and the duodenal mucosal-to-serosal flux of permeability markers were not statistically different between experimental groups (Supplementary Table S4).

### Microbial Community Profiles – Sampling Site Comparison

Sequencing of pig samples (feces-start, feces-end, ileum, ICLN) resulted in 18,311,300 reads. After quality control, 16,794,141 reads (91.7%) remained, clustering into 5,394 operational taxonomic units (OTUs; 0.03 distance level). In total, 43 OTUs were identified as potential contaminants and summed up in a list (Supplementary Table S5). These OTUs were generally of low prevalence and not part of the OTUs discussed in the following sections.

**FIGURE 1:**
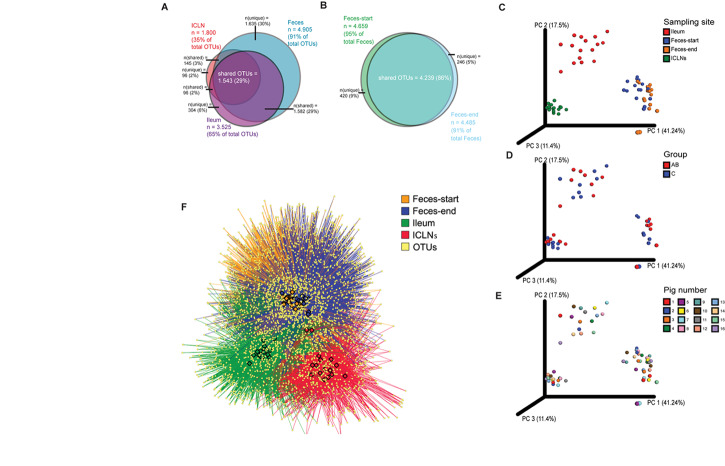
Beta diversity analysis of microbial communities. Overlaps of OTUs detected in **(A)** the three sample groups (Feces, ileum, ICLNs), and in **(B)** Feces-start and feces-end. Weighted UniFrac distances based on 16S rRNA gene libraries. Each point represents values from individual libraries with colors expressing **(C)** Ileum, Feces-start, Feces-end, and ileocaecal lymph node (ICLN) samples; **(D)** Control and antibiotic group samples (Feces-start samples were not separated into antibiotic-treated (AB) and control groups and are therefore not shown here); and **(E)** Pig samples. **(F)** OTU network of bacterial communities. Individual OTUs are represented by light yellow dots. Colored edges connect OTUs with the respective tissue in which they were detected: Ileum (red), Feces-start (blue), Feces-end (orange), ICLN (green). Colored nodes embody individual samples (same color code as edges). OTUs in the center of the network are shared among tissues, whereas OTUs on the outer rim are specifically present in certain tissues.

Alpha diversity indices differed significantly between all sampling sites except for the Simpson’s index of diversity, which showed a significant difference between ileum and feces-start/end as well as Ileum and ICLN samples, but not between feces-start compared to feces-end and feces-start/end compared to ICLN samples. Fecal samples showed the highest diversity with more than 1,900 observed OTUs, while ileum and ICLN samples harbored more than 1,100 and more than 350 observed OTUs, respectively (Table 1). Overall, 29% of all OTUs were shared between the sampling sites, whereas feces-start and feces-end samples had a more consistent community structure, sharing 86% of their OTUs (Figure 1A,B). Weighted UniFrac results show a strong separation of different sampling sites suggesting that the overall composition of the microbial community was largely affected by sampling site (Figure 1C). In contrast, microbiota of antibiotic treated pigs compared to control pigs and individual pigs compared to each other dispersed only slightly from each other (Figure 1D,E). An OTU network analysis achieved a more precise view on the OTU distribution across samples (Figure 1F).

**Table 1:** Diversity estimates of microbial communities.

		Mean ± SD			
		
Group	Tissue	Observed OTUs	Chao1	Shannon	Simpson
Control	Feces-start	2225 ± 194.54	2889.58 ± 156.99	4.38 ± 0.27	0.94 ± 0.03
	Feces-end	2085.38 ± 318.54	2784.42 ± 381.64	4.04 ± 0.6	0.92 ± 0.03
	Ileum	1101.75 ± 161.93	1611.59 ± 166.33	2.59 ± 0.89	0.77 ± 0.23
	ICLN	436.88 ± 176.75	759.28 ± 270.57	3.55 ± 0.21	0.89 ± 0.04
AB	Feces-start	2365.13 ± 279.28	2998.69 ± 226.2	4.36 ± 0.58	0.93 ± 0.04
	Feces-end	1900 ± 213.57	2513.66 ± 260.61	3.84 ± 0.63	0.91 ± 0.05
	Ileum	1165.75 ± 197.66	1716.42 ± 271.46	2.94 ± 0.57	0.84 ± 0.8
	ICLN	361.5 ± 184.04	617.12 ± 291.39	3.52 ± 0.25	0.89 ± 0.05

		***p*-value**			

Ileum – Feces-start		< 0.0001	< 0.0001	< 0.0001	0.0017
Ileum – Feces-end		< 0.0001	< 0.0001	< 0.0001	0.0055
Ileum – ICLN		0.0297	0.0009	0.0884	0.0203
Feces-start – Feces-end		0.0297	0.0164	0.0358	0.5315
Feces-start – ICLN		< 0.0001	< 0.0001	< 0.0001	0.3156
Feces-end – ICLN		< 0.0001	0.0343	< 0.0001	0.5315
Feces-end AB – Feces-end C		0.2025	0.1422	0.4722	0.8638
Ileum AB – Ileum C		0.3638	0.2316	0.4482	0.3710
ICLN AB – ICLN C		0.8695	0.941	0.989	0.9707


*Absolute values represent data from the full dataset. p-values were calculated with the rarefied dataset. SD, Standard deviation; AB, Antibiotic group; C, Control group; ICLN, Ileocecal lymph node*.

**FIGURE 2:**
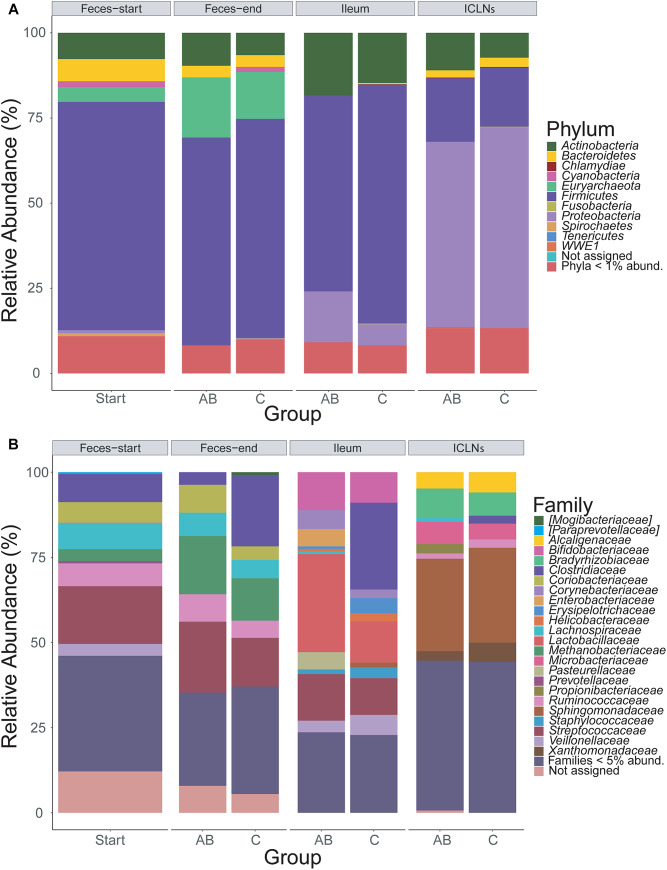
Taxonomic classification of 16S rRNA gene sequence reads. Taxonomic classification of 16S rRNA gene sequence reads parted by sampling site and group (AB or control). Data represents average of OTU counts from replicate libraries for each category. **(A)** Phylum-level classification. **(B)** Family-level classification. Phyla with less than 1% and families with less than 5% relative abundance (abund.) were grouped together. Sequences that could not be assigned are depicted as “Not assigned.” Start = Feces samples taken prior to antibiotic treatment (Thus not divided in AB and C group), AB = Antibiotic group, C = Control group, ICLN = Ileocecal lymph nodes.

Throughout all samples, 24 phyla were identified with *Firmicutes, Proteobacteria*, and *Actinobacteria* being the most abundant ones (Figure 2A). The overall phyla abundance composition differed significantly between sampling sites (Table 2; *p* < 0.001). In detail, feces and ileum samples were dominated by *Firmicutes*, whereas ICLN samples harbored mostly *Proteobacteria. Bacteroidetes* and *Euryarchaeota* were more abundant in fecal samples compared to ileum and ICLN samples (Supplementary Table S6; Spreadsheet “Phyla”).

**Table 2:** PERMANOVA test for the influence of sampling site, group, and both interdependently.

	PERMANOVA	*p*-value	*R*^2^	Betadispersion permutest (*p*-value)
Phylum	Sampling site	<0.001	0.750	NS
	Group	0.362	0.004	NS
	Sampling site:Group	0.482	0.011	NS
Family	Sampling site	<0.001	0.571	<0.001
	Group	0.012	0.021	<0.001
	Sampling site: Group	0.252	0.025	<0.001
OTU	Sampling site	<0.001	0.467	<0.001
	Group	0.037	0.018	<0.001
	Sampling site: Group	0.017	0.045	<0.001


*Results from a PERMANOVA test for the influence of sampling site (feces-start, feces-end, ileum, or ICLN), group (AB or control), and both interdependently on the differences in community structure. PERMANOVA = permutational analysis of variance; ICLN = ileocecal lymph node; AB = antibiotics; NS = Not significant*.

Family-level analysis identified 144 families with 24 major families with relative abundances over 5% in at least one sample (Figure 2B and Supplementary Table S6; Spreadsheet “Families”). The composition of family abundances differed significantly between sampling sites (Table 2; *p* < 0.001), whereas this result is influenced not only by the differences in composition between sampling sites but also by the difference within sampling site groups (*p* < 0.001). The majority of the samples showed a homogenous family-level profile within each sampling site, but a few samples exhibited remarkable differences, mainly due to a considerable increase in abundance of one or two OTUs (Supplementary Table S6).

Comparing feces-start to feces-end samples we found that *Methanobacteriaceae* were enriched (*p* < 0.006), whereas *Veillonellaceae* were comparatively reduced (*p* < 0.004) at the end of the experiment. The increase of *Methanobacteriaceae* was particularly attributed to the second most abundant OTU across all samples, which was assigned to the genus *Methanobrevibacter*. Several other highly abundant OTUs showed significantly different relative abundances in feces-start or feces-end samples. These were assigned to *Lactobacillus, Mitsuokella, Treponema, Ruminococcaceae, Coriobacteriaceae*, and other taxa.

A comparison of relative abundances of microorganisms in ileum samples to ICLN samples revealed substantial differences between the two habitats. For example, ICLNs were dominated by *Microbacteriaceae, Ruminococcaceae, Bradyrhizobiaceae, Sphingomonadaceae, Alcaligenaceae*, and *Xanthomonadaceae*, whereas ileum samples were primarily composed of *Lactobacillaceae, Bifidobacteriaceae, Streptococcaceae*, and *Clostridiaceae*. Detailed results can be found in Supplementary Table S6; Spreadsheet “Families” and “OTUs.”

### Effect of Antibiotics on the GI Microbiota

Medicated feed did not have a significant effect on alpha diversity indices in the analyzed groups (Table 1). However, a permutational analysis of variance (PERMANOVA) exposed the factor “group” (AB or control) to be a significant source of variation across all samples. Additionally, our findings determine a variability within the groups which influenced the ascertained variation across the samples (Table 2). Hence, we identified specific phylotypes whose abundance varied significantly between antibiotic and control treatment at different taxonomic levels (Supplementary Table S6).

At the phylum level we did not observe a significant difference between the two treatment groups (*p* = 0.362). Still, five low abundant phyla were diminished in the AB group of ileum or fecal samples (e.g., *Chlamydiae, Chloroflexi, Cyanobacteria* (lineage YS2, reclassified to *Melainabacteria*), *Elusimicrobia*, and *Fibrobacteres*).

The family abundance composition differed significantly between treatment groups (Table 2; *p* = 0.012; detailed comparison in Supplementary Table S6; Spreadsheet “families”). In the control group, feces-start and feces-end samples did not differ significantly in any abundant families (>1% relative abundance; *p* > 0.05), indicating a stable microbial community profile over the course of the experiment. In the AB group, *Methanobacteriaceae* were enriched (7.63-fold change), and *Veillonellaceae* were reduced (5.02-fold change) in feces-end samples compared with feces-start samples (*p* = 0.021 and *p* = 0.014, respectively). At the ileal mucosa, *Clostridiaceae, Chlamydiaceae*, and *Halomonadaceae*, were significantly reduced in the AB group (49.01-fold change, 15.83-fold change, and not observed, respectively).

**FIGURE 3:**
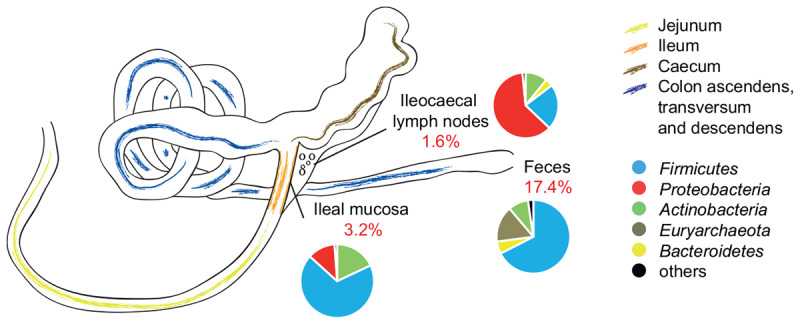
Schematic drawing of the pig gastrointestinal (GI) tract anatomy. Pie charts represent mean relative abundances of 16S rRNA gene sequences associated to phylum level taxonomy. Percentage values in red depict proportion of OTUs that were significantly affected by AB treatment.

Also at OTU level, the abundance composition revealed significant differences between groups (Table 2; *p* = 0.037). The biggest shifts in microbial abundance induced by antibiotics were observed in fecal samples. In detail, antibiotic treatment had a significant impact on 780 (out of 4,485, 17.4%) OTUs in feces-end, 114 (out of 3,525, 3.2%) OTUs in ileum, and 29 (out of 1,800, 1.6%) OTUs in ICLN samples compared to the control group (Figure 3).

Consistent to family level analysis, we compared OTU abundances in feces-start with feces-end samples in each group. In the control group feces-start and feces-end samples did not differ significantly among the 50 most abundant OTUs (*p* > 0.05). In the AB group, feces-end samples were significantly enriched with a highly abundant *Methanobrevibacter*- (*p* = 0.0048, 20.17-fold change) and a *Ruminococcus*-affiliated OTU (*p* = 0.0077, 134.82-fold change) and reduced in a highly abundant *Clostridiaceae*-affiliated OTU (*p* = 0.0012, 104.91-fold change) (Supplementary Table S6; Spreadsheet “OTUs”).

**FIGURE 4:**
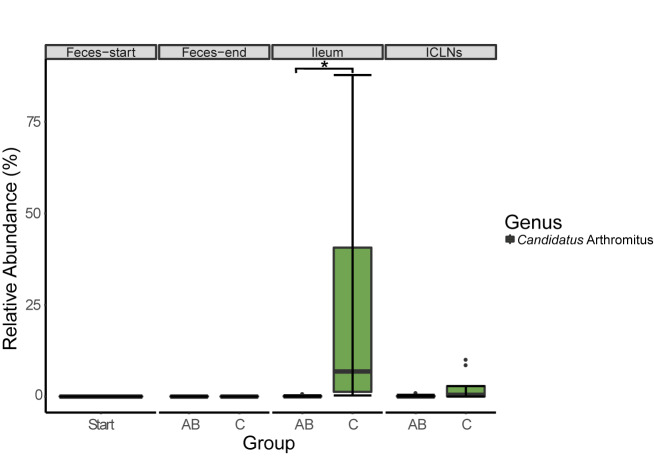
Relative abundance of 16S rRNA gene sequences associated to *Candidatus* Arthromitus. Boxes indicate the interquartile range (75th to 25th) of the data. The median value is shown as a line within the box. Whiskers extend to the most extreme value within 1.5 × interquartile range. Significance code: ^∗^*p* = 0.0479.

At the ileal mucosa, two prevalent OTUs belonging to the genera “*Candidatus* Arthromitus” (*p* = 0.048, 199.17-fold change, Figure 4) and *Sharpea* (*p* = 0.025, 160.45-fold change) have been identified to be lower abundant in ileal samples of the AB group compared to the control group. A BLAST search against the NCBI nucleotide collection revealed that this OTU showed 98% sequence identity to *Candidatus* Arthromitus and 100% sequence identity to an uncultured bacterium belonging to the SFB group-7, previously detected in piglet intestines (unpublished; Accession number: AB822985.1).

In ICLNs, one of the 50 most abundant OTUs, associated with the family *Helicobacteriaceae*, was significantly decreased in the AB group of ICLNs compared to the control group (*p* = 0.026, 6.08-fold change).

### Metabolically Active Microorganisms in ICLNs

In the cultivation approach, 20 bacterial species were isolated from ICLNs. The isolates were assigned to 18 genera, including *Escherichia* (51.40%), *Streptococcus* (14.02%), and *Corynebacterium* (9.35%) as the most abundant ones (Supplementary Table S7). Additionally, several genera were represented by a single isolate, e.g., *Pantoea agglomerans* and *Dietzia aurantiaca* which have not been associated with pig intestines until now. All *Escherichia* isolates were subjected to a multiplex PCR for the detection of virulence genes. No shiga-toxin producing *E. coli* (STEC) was detected, but six isolates were positive for the gene *eae*, coding for Intimin, a protein of enteropathogenic *E. coli* (EPEC) involved in the attachment to epithelial cells (Jerse et al., 1990). The BLASTn comparison of isolates with Illumina sequence representative OTUs revealed 14 hits with >250 bp alignment length, among these were 3 bacilli, four Corynebacteria as well as the unexpected microorganisms *Dietzia* and *Pantoea* (Supplementary Table S8).

**FIGURE 5:**
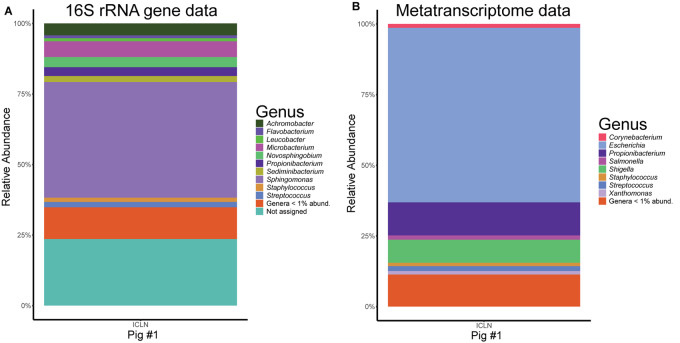
Comparison of the microbial community composition in ICLNs from pig #1. **(A)** Relative abundances of 16S rRNA gene sequences. **(B)** Relative abundance of metatranscriptome reads. Genera with less than 1% relative abundance (abund.) were grouped together.

We analyzed the metatranscriptome of a single ICLN sample to gain preliminary insights on the functional potential of the ICLN-associated microbiome. After removal of artificial replicate sequences and host specific sequences, 15,527 sequences of bacterial origin were annotated to the KEGG orthology database. Similar to the 16S rRNA gene profiling, *Proteobacteria* (78%) and to a lesser extent *Actinobacteria* (17%) and *Firmicutes* (4%) made up the vast majority of the sequences. Genus-level association revealed that 61% of the sequences were assigned to *Escherichia* and not to *Sphingomonas* as depicted by the 16S rRNA gene community profiles (Figure 5). Intriguingly, other highly abundant genera in the metatranscriptome dataset were, e.g., *Propionibacterium* (12%), *Streptococcus* (2%), and *Corynebacterium* (1%), which is mostly congruent with isolates from the cultivation approach. In total, we identified 269 genera and 153 families with the metatranscriptome approach. Table 3 provides an overview of the functional features of all annotated sequences and an additional file shows which genera were mapped to the most prevalent functional features (Supplementary Table S9). A large part of the sequences was associated with general metabolic features like amino acid and carbohydrate metabolism as well as energy, nucleotide, and lipid metabolism. More detailed analysis showed the presence of transcripts for a complete citrate cycle and glycolysis pathway, although we are not able to show that these originate from a single genome. Furthermore, many sequences were related to membrane transport and signal transduction, further indicative of an active community. Additionally, we detected sequences linked with DNA replication, translation, and protein folding and degradation, revealing an actual turnover of proteins and possibly even growth of bacteria in ICLNs.

**Table 3:** Metatranscriptome sequences associated to functional features obtained from one ICLN sample.

Associated functional domain	Relative abundance of sequences [in %]
Amino acid metabolism	17.72
Carbohydrate metabolism	12.54
Membrane transport	12.02
Translation	7.68
Metabolism of cofactors and vitamins	7.41
Signal transduction	6.97
Replication and repair	6.10
Nucleotide metabolism	4.74
Energy metabolism	4.56
Lipid metabolism	3.29
Folding, sorting and degradation	3.25
Glycan biosynthesis and metabolism	2.72
Membrane transport	1.71
Metabolism of terpenoids and polyketides	1.40
Transport and catabolism	1.23
Cell growth and death	1.14
Transcription	1.14
Cell motility	1.10


*Features over 1% relative abundance are shown*.

## Discussion

The GI microbiome represents a highly complex ecosystem with a large potential to influence host health. The close connection between the mucosa-associated microbiota and lymph nodes is a driving factor behind bacterial-triggered host immune activation. It is well known that an altered gut microbiome has outstanding effects on the mammalian immune system (Palm et al., 2016; Laforest-Laponte and Arrieta, 2017), but remarkably little is known about the actual role of the lymph node-associated microbiome. This study provides the first combined community profiling survey of fecal, ileal and ICLN-associated microorganisms. We investigated the influence of Linco-Spectin and Colistin sulfate on the microbiome of these different sampling sites, increasing the knowledge about antibiotic effects on bacterial communities in an *in vivo* situation.

The bacterial/archaeal primer pair chosen (515F-806R) targets the V4 region of the 16S SSU rRNA gene and is traditionally used by the Earth Microbiome Project (The Earth Microbiome Project/Protocols and Standards). It was shown that it performs well in archaeal detection with a known bias against *Crenarchaeota/Thaumarchaeota* (Walters et al., 2015). As, we do not expect these phyla in the pig’s GI tract, we did not filter out archaeal reads and analyzed them together with bacterial reads. Furthermore, the importance of archaea within the intestinal microbiota and gut physiology has been underestimated for a long time but has received increased awareness in the last few years (Nkamga et al., 2017). Vierbuchen et al. (2017) revealed a specific recognition and response to the archaeon *Methanosphaera stadtmanae* by human immune cells (Vierbuchen et al., 2017), demonstrating that archaea represent an important part of the intestinal microbiota and should therefore be included in future gut microbiome studies.

We could identify characteristic antibiotics-associated shifts in the microbiome at the various intestinal sampling sites including the ileal mucosa. By contrast, gut electrophysiological measurements indicated that the mucosal integrity and ion secretion in the mid jejunum was not altered by the antibiotics, as indicated by the similar jejunal G_T_ and I_SC_, as indicators for paracellular permeability and total ion movements (Clarke, 2009), between the two pig groups. So far, physiological gut permeability alterations have been associated with weaning, chronic stress, dietary changes and GI disturbances, but little is known about the impact of antibiotics on gut permeability in pigs. In using a transcriptomics-based approach in chickens, for instance, Schokker et al. (2017) reported an upregulation of tight-junction-protein expression in the jejunum of birds fed amoxicillin from early life onward and associated this with the observed perturbations in the jejunal microbiota. Therefore, it might have been assumed that the present longterm administration of antibiotics via the feed may have caused similar alterations in the jejunal barrier function. Regardless of the animal species and potential different effects of the various types of antibiotic administrated, however, it needs to be considered that we sampled a small gut section in the mid-jejunum and therefore only have the local impact of the antibiotics for this gut site. As the antibiotics were orally administrated, effects may therefore occur in more proximal or distal parts of the GI tract. Moreover, the effect of antibiotics is expected to differ between classes of antibiotics, whereby permeability alterations did not always result from microbiota shifts and vice versa (Tulstrup et al., 2015). In addition, in assuming that gut microbiota shifts due to antibiotics are behind alterations in the gut permeability, the activity spectrum of Linco-Spectin and Colistin sulfate should be considered as the jejunal community may have been less affected by the antibiotics, leading to similar microbe-host-interactions in the jejunum, compared to the investigated ileal community. Linco-Spectin is a combination of Lincomycin and Spectinomycin, has a bacteriostatic effect, and targets protein synthesis by binding to bacterial ribosomes. Its antimicrobial activity is mainly directed toward *Staphylococcaceae, Streptococcaceae, Corynebacteriaceae, Erysipelothrix, Leptospiraceae*, and *Clostridiaceae*. Colistin sulfate belongs to the group of polymyxins and is bactericidal. Its antimicrobial activity is mainly directed toward phospholipid components of the cytoplasmic membrane and the lipopolysaccharide, increasing the permeability of the cell membrane, thus leading to the leakage of cell contents and eventually cell death. It is especially effective against *E. coli, Salmonella, Shigella, Vibrio*, and *Yersinia* (Riviere et al., 2009), occurring at higher numbers in the ileum than in the jejunum of pigs (Kelly et al., 2017). Therefore, potential gut permeability-related changes along the GI due to Linco-Spectin and Colistin sulfate medicated-feed warrants further investigation. With the combination of these two antibiotics we aimed to cover a broad spectrum of antimicrobial activity. Collectively, the used antibiotics led to a reduction in relative abundance of *Clostridiaceae* and several members of the families *Coriobacteriaceae, Erysipelotrichaceae, Helicobacteriaceae*, and *Staphylococcaceae*. We could not observe any effect against *Streptococcaceae* or *E. coli*, which were also among the targeted groups. In contrast, relative numbers of *Streptococcaceae* were higher in the AB group, although this increase was not significant. It is likely that the AB treatment caused a reduction of absolute numbers of total bacteria, thereby masking smaller effects on specific bacterial taxa. However, most of the reduced bacteria are known to be susceptible to the used antibiotics.

### Microbial Community Profiles – Sampling Site Comparison

Phylum level profiles of 16S rRNA gene libraries of fecal, ileum and ICLN samples largely resembled the microbial composition in pig intestines of previous studies with *Firmicutes, Bacteroidetes, Proteobacteria*, and *Actinobacteria* dominating in the gut, and *Proteobacteria* strongly dominating in ICLNs (Dowd et al., 2008; Isaacson and Kim, 2012; Looft et al., 2014; Mann et al., 2014a,c). Alpha and beta diversity analysis unveiled extensive differences between the fecal, the ileal, and the ICLN-associated microbiome in pigs. The ICLN microbiome represents a sub-fraction of the mucosal gut microbiome with a significantly lower diversity compared to ileum and feces. In total, 98% of the OTUs detected in ICLNs were also found in the ileum or in the feces. This is expected, since microorganisms living close to the intestinal epithelium represent a possible inoculum for the ICLN microbiome. However, the abundance patterns differ at these two sampling sites, which might be explained by (i) differences in nutrient availability (Hibbing et al., 2010), (ii) individual bacterial survival times and bacteria that are able to thrive in ICLNs, which subsequently leads to an enrichment (Nagl et al., 2002; Macpherson and Uhr, 2004), (iii) time-related differences in immune cell processing (Macpherson and Uhr, 2004), and/or (iv) selective sampling of bacteria by immune cells (Morikawa et al., 2016). Phylotypes that were highly abundant in feces (e.g., *Ruminococcaceae*), but not at the ileal mucosa, were detected in high abundance in ICLNs, suggesting selective sampling of immune cells in the gut lumen. Here, we demonstrate that *Proteobacteria* make up the majority of the microbial community in ICLNs. They have also been found to be the most abundant phylum in other lymph node locations as well as in lymph nodes of other mammals (Wittekindt et al., 2010; Mann et al., 2015a). Together with our data, this suggests that *Proteobacteria* might constitute the physiological microbiota of lymph nodes, potentially independent of host species and localization. Family level classification revealed *Sphingomonadaceae* at high abundances in all ICLN samples with the DNA-based 16S rRNA gene sequencing approach. However, they were detected to a much lesser extent with the cultivation and metatranscriptomic techniques. *Sphingomonadaceae* possess glycosphingolipids instead of lipopolysaccharides in their cell envelopes which are presented to invariant natural killer T cells by a MHC class I-like molecule expressed by professional antigen presenting cells (APCs), including DCs in ICLNs (Van Kaer and Joyce, 2005; Matsuda et al., 2008; Rossjohn et al., 2012). Activation of invariant natural killer T cells by commensal bacteria is an important part of regulating the immune system and *Sphingomonas* species have been recognized to take part in that (Wei et al., 2010; Olszak et al., 2012; Brestoff and Artis, 2013). Consequently, we hypothesize that they are immediately eliminated by the immune system upon transmission to lymph nodes. Hence, this would explain the discrepancy in *Sphingomonadaceae* abundance between the DNA-based 16S rRNA gene sequencing data, which can also detect DNA from dead organisms, and the RNA-based metatranscriptome data.

At the ileal mucosa, *Lactobacillaceae, Bifidobacteriaceae, Clostridiaceae*, and *Streptococcaceae* were detected at high abundances. *Lactobacillaceae* and *Bifidobacteriaceae* are known to attach to the mucosal epithelium of vertebrates and exert a number of beneficial effects on the host, e.g., enhancement of the intestinal epithelial barrier integrity, modulation of immune responses, and competitive exclusion of pathogens (von Ossowski et al., 2010; Pessione, 2012; Sengupta et al., 2013; Turroni et al., 2013).

The *Clostridiaceae* family was highly abundant in ileal and ICLN samples. Overall, 30.04% of all reads belonging to this family were affiliated to the genus “*Candidatus* Arthromitus,” which was represented by one of the most abundant OTUs in ileal samples. “*Candidatus* Arthromitus” is a member of the segmented filamentous bacteria (SFB) with its taxonomic position still being under debate. The proposed name “*Candidatus* Savagella” for mammalian-associated SFB gut symbionts (Thompson et al., 2012) is not in current databases, wherefore we used “*Candidatus* Arthromitus” throughout this manuscript. “*Candidatus* Arthromitus” have undergone a close symbiotic relationship with their host, where they are commonly detected at the ileal mucosa (Ericsson et al., 2014; Holman et al., 2017). Their ability to induce host immune responses without actually having pathogenic potential conveyed them increased attention in various research fields in the last few years (Kuwahara et al., 2011; Prakash et al., 2011; Sczesnak et al., 2011). They induce Th-17 cells through modulation of DC activity via specific ADP- ribosyl transferases (Pamp et al., 2012; Goto et al., 2014; Farkas et al., 2015). Thus, they are thought to play an important role in the development of multiple adaptive immune responses (Schnupf et al., 2017).

Fecal samples harbored *Euryarchaeota*, which were comprised mainly of a specific OTU associated with the genus *Methanobrevibacter*, that has been detected in piglet feces in the past (Su et al., 2014; Federici et al., 2015). *Methanobrevibacter* utilizes hydrogen, as well as acetate and formate as electron donors to reduce carbon dioxide to methane, what might affect energy metabolism in terms of energy loss as reported in cattle (Johnson and Johnson, 1995). Despite of high *Methanobrevibacter* abundances in the feces, the ileal mucosa and also ICLNs harbored only few *Methanobrevibacter*, indicating that the lower GI tract is an exclusive niche. Concurrently, a proximal to distal gradient of methanogenic archaea along the intestinal tract has been reported by others as well (Macfarlane et al., 1992; Nava et al., 2012). Cellulolytic bacteria like *Ruminococcus* showed a similar pattern being most abundant in the fecal samples.

### Microbial Community Profiles – Group Comparison

Since the pigs had free access to the feeding troughs, it is likely that some pigs consumed more feed than others. However, the overall weight gain of most of the pigs was very similar, indicating that the general food intake was alike among pigs, but we do see two outliers in weight gain, one with little and one with high weight gain, which possibly also involves over- or underdosing of antibiotics for these pigs. Family and OTU level-, but not phylum level association exposed significant differences between the AB and control group. Recently, this was also described for in-feed antibiotics (e.g., monensin and tylosin) (Thomas et al., 2017), indicating an overall stable microbial profile but profound effects on the distribution patterns of phylotypes at lower taxonomic levels.

In the ileum, “*Candidatus* Arthromitus,” a member of SFB and one of the most abundant OTUs in our dataset, was found to be significantly diminished in the AB group. SFB have been connected to pathogen protection in numerous cases (Heczko et al., 2000; Burgess et al., 2014). Their capability to induce Th-17 cells has also been shown to drive autoimmunity in mice that were monocolonized with SFB (Lee et al., 2011; Teng et al., 2016). Thus, the role of SFB in host immune response is not fully understood yet and further research has to be done to investigate this delicate relationship. Our data advocates that SFB are highly susceptible to Colistin sulfate and Linco-Spectin. In mice, it has been reported that SFB levels have not recovered even 3 weeks after antibiotic treatment with broad-spectrum coverage (Croswell et al., 2009). Although the barrier function of the small intestine was not disturbed by the antibiotics used, we hypothesize that with the sudden, severe reduction of SFB along the mucosal epithelium upon antibiotic treatment, the immune system suddenly loses an important beneficial stimulus and pathogenic bacteria attach and invade the intestinal epithelium more efficiently, as SFB cannot secure their beneficial effects at the mucosa. The host might be more prone to recurrent infections even weeks after antibiotic therapy.

In this study, the used antibiotics also effectively reduced the amount of *Chlamydiae* in ileum samples. All sequences affiliated to the phylum *Chlamydiae* showed 100% sequence identity with *Chlamydia (C.) suis*, indicating an asymptomatic *C. suis* infection as reported before in pigs (Schautteet and Vanrompay, 2011; Li et al., 2017). *C. suis* infections occur globally and can be related to enteritis, pneumonia, reproductive failure, and conjunctivitis (Schautteet and Vanrompay, 2011; Li et al., 2017). Recently, *C. suis* DNA has also been detected in pharyngeal and rectal swabs of pig farmers, indicating transfer of *C. suis* from pigs to humans that had direct contact with infected pigs (De Puysseleyr et al., 2017). The zoonotic potential of *C. suis* is concerning with reports about tetracycline resistant strains accumulating in the last few years (Francesco et al., 2008; Schautteet et al., 2013; Li et al., 2017). With that in mind, it is important to note that Colistin sulfate and Linco-Spectin might be possible alternatives for *C. suis* treatment in case of GI infections.

In fecal samples, *Methanobrevibacter* numbers increased over the course of the experiment in all pigs (feces-start vs. feces-end, *p* = 0.003), which can be explained by an ongoing continuous maturation of the microbiota during the experiment. However, separated by groups, only the AB groups, but not the control group, showed a significant increase (AB group, *p* < 0.005; control group, *p* = 0.314; Supplementary Table S6 Spreadsheet “OTUs”). Thus, we believe that *Methanobrevibacter* was able to quickly occupy freed niches from bacteria that were effectively killed by the antibiotics. Since it competes with sulfate reducing bacteria and with acetogens for H_2_, the metabolization of H_2_ might be different in antibiotic-fed pigs compared with pigs from the control group with a possible increase in methane production in the AB group.

Also, a *Ruminococcus*-affiliated OTU was significantly enriched in the feces of AB group pigs. *Ruminococcus* species have been associated to increased weight gain and higher fat content in pigs (He et al., 2016; Fang et al., 2017). Another OTU related to *Sharpea* was found to be significantly diminished in the AB group of fecal samples. *Sharpea* has previously been associated with increased lactate formation and low-methane emission in sheep (Kittelmann et al., 2014; Kamke et al., 2016). Thus, our antibiotic treatment favored methane-producing species and effectively reduced species that are able to mitigate methane emissions. Accordingly, Hammer et al. (2016) observed a positive correlation of antibiotic treatment and methane emissions in cattle dung (Hammer et al., 2016). In the past, research on antibiotics has been focused strictly on their direct effects within the animal or human body, but it is conceivable that changes of microbial communities in the intestinal tract of livestock upon antibiotic treatment also affect global biogeochemical cycles. While we can only hypothesize on that with the data presented here, these findings offer an interesting stepping stone for future research.

Collectively, the used antibiotics were effective against *Clostridiaceae* and several members of the families *Coriobacteriaceae, Erysipelotrichaceae, Helicobacteriaceae*, and *Staphylococcaceae*. We could not observe a significant effect against *Streptococcaceae*, which was among the targeted groups. *E. coli* was also not significantly reduced, which might be caused by already very low numbers of *Enterobacteriaceae* at the beginning of the study (feces-start, median relative abundance: 0.02%).

### Metabolically Active Microorganisms in ICLNs

In this study, a broad range of cultivation media was used under aerobic and anaerobic conditions. However, strictly anaerobic microorganisms might be missed due to interruptions of the anaerobic environment during sampling procedures (e.g., oxygen invasion of lymph nodes during dissection) and processing. Overall, we managed to obtain several isolates of *Escherichia, Streptococcus* and *Corynebacterium* species from various different pigs, suggesting that they represent a large fraction of the core microbiome of ICLNs. Especially *E. coli* is thought to be prevalent in lymphatic tissues and has already been detected numerous times in the past (Berg and Garlingtont, 1980; Wells et al., 1987; Sechi et al., 2012; Mann et al., 2015a). Six *E. coli* isolates were positive for the bacterial surface protein Intimin (encoded by the *eae* gene). Enteropathogenic *E. coli* (EPEC), are defined by encoding *eae* but not *stx* (An et al., 1997) and were detected in ICLNs of pigs before (Mann et al., 2015a), showing the great potential of these pathogenic bacteria to survive in ICLNs.

*Streptococcus alactolyticus* is a widely distributed lactic acid bacterium which is commonly found in pig intestines. The isolated *Corynebacterium* (*C*.) strains were mostly non-pathogenic except for *C. xerosis* and *C. stationis. C. xerosis* has undergone increased concerns about its zoonotic significance lately (Vela et al., 2006; Palacios et al., 2010). *C. stationis*, previously known as *Brevibacterium stationis*, has also been isolated from blood cultures obtained from patients with chest infections (Bernard et al., 2010; Bernard, 2012). In addition to these prevalent species, we also acquired single isolates of 14 species, which we did not expect to be viable in ICLNs: e.g., *P. agglomerans*, an opportunistic human pathogen usually associated with plants, that has been isolated from numerous different environments as well (Walterson and Stavrinides, 2015), or *Dietzia aurantiaca*, which was just recently described as a new species isolated from human cerebrospinal fluid (Falsen et al., 2012). Both organisms had 100% similarity to the representative OTU sequence in the Illumina dataset. To our knowledge, these bacteria were isolated for the first time in porcine ICLNs.

We also sequenced the metatranscriptome of a single ICLN and detected 269 different genera, suggesting a high diversity of viable bacteria in ICLNs. However, 120 of the genera had only 1–2 sequences assigned to them. These sequences might be false-positives due to wrong assignment, horizontal gene transfer, or they are leftover mRNA of dead bacteria that has not been fully degraded yet. Thus, a larger cohort has to be sequenced to determine whether these bacteria are really metabolically active in lymph nodes or not. Overall, the metatranscriptome data, although similar to the cultivation data, shows contradictory results when compared to the 16S rRNA gene sequencing data (Figure 5). In principal, one would not expect consistent results between these two approaches, since DNA-based 16S rRNA gene community profiling does not differentiate between dead or viable bacteria and we do expect a high number of already processed or inactivated antigens in ICLNs. In this case, 16S rRNA gene profiling revealed *Sphingomonadaceae* as the most abundant family whereas the majority of the sequences of the metatranscriptome were associated with *E. coli*. In addition, the majority of the isolates obtained via cultivation were also identified as *E. coli*. Apparently, *E. coli* makes up only a small part of the microbial community in porcine ICLNs but it is still the most active one. The fact that it is metabolically active is concerning, because it is used as an indicator of food hygiene and regularly contaminates pork carcasses (Biasino et al., 2018). Hence, lymph nodes should be considered as a reservoir of pathogenic bacteria, making them a risk hazard for food safety and spoilage. This is all the more important, because our results demonstrate that the antibiotics used, are not threatening bacteria residing in ICLNs. Thus, we believe that the activity of the microbiota will largely stay equal in response to antibiotic treatment, although further research is necessary to investigate these effects at this juncture. Even if metatranscriptome data is limited in this study, this data indicates that ICLNs have an active and diverse microbial community, comparable to other lymphatic tissues previously studied by our group (Mann et al., 2015b).

Pigs are used as model organisms and have physiological and immunological similarities to humans. Therefore, our study provides important insights into the complex interplay between the gut-lymph node microbiota. The ICLN microbiome represents a sub-fraction of the ileal microbiome. Differences in bacterial enrichment patterns at these two sampling sites can be explained by differences in nutrient availability, individual bacterial survival times, delayed immune cell processing, or selective sampling of bacteria.

Our results indicate that pathogenic bacteria (e.g., EPEC) could escape antibiotic treatment, if they are translocated to lymph nodes, making the ICLN-associated microbiota a central element for bacteriological risk analysis and the understanding of cross-contamination during slaughter of livestock. This is especially significant, because one of the main risk factors during slaughtering and meat cutting is the transmission of bacteria that derive from the GI tract or its adnexa (e.g., lymphatic tissues) (Rostagno, 2009).

We identified SFB that are associated with adjuvant host immune responses, to be susceptible to antibiotic treatment along the ileal mucosal epithelium. Thus, we believe that SFB might play an important role in antibiotic-induced dysbiosis in farm animals. Further studies are necessary to investigate their exceptional role in actuating host immune responses. Finally, antibiotic treatment increased *Methanobrevibacter*, and decreased *Sharpea* relative abundances, which could potentially lead to higher methane emissions of livestock.

## Author Contributions

EM, BM-Z, MW, and AL were involved in study design. EM, BM-Z, BZ, CK, SW, and MH conceived the experiments and BZ, BP, EM, MH, KG, MD, SS-E, and BM-Z analyzed the data. BP conducted the statistical analysis. BZ, EM, BP, MD, and BM-Z wrote the manuscript. All authors approved the final manuscript.

## Conflict of Interest Statement

The authors declare that the research was conducted in the absence of any commercial or financial relationships that could be construed as a potential conflict of interest.
