# Erratum: Microbiota of the Gut-Lymph Node Axis: Depletion of Mucosa-Associated Segmented Filamentous Bacteria and Enrichment of *Methanobrevibacter* by Colistin Sulfate and Linco-Spectin in Pigs

**DOI:** 10.3389/fmicb.2020.01051

**Published:** 2020-05-26

**Authors:** Frontiers Production Office

**Affiliations:** Frontiers Media SA, Lausanne, Switzerland

**Keywords:** antibiotics, lymph nodes, ileum, microbiome, 16S rRNA gene, metatranscriptome, segmented filamentous bacteria, gut microbiota

Due to a production error, the Data Availability Statement was not included in the article. The Data Availability Statement appears below. The publisher apologizes for this mistake.

“16S rRNA gene sequencing data and metatranscriptome data were submitted to the European Nucleotide Archive (ENA) with the accession number PRJEB15104.”

The original article has been updated.

