# Supplementary figures and images for: Microbiota of the Gut-Lymph Node Axis: Depletion of Mucosa-Associated Segmented Filamentous Bacteria and Enrichment of Methanobrevibacter by Colistin Sulfate and Linco-Spectin in Pigs
Source: Front Microbiol. 2019 Apr 11;10:599. doi: 10.3389/fmicb.2019.00599 (PMC6470194; doi:10.3389/fmicb.2019.00599)

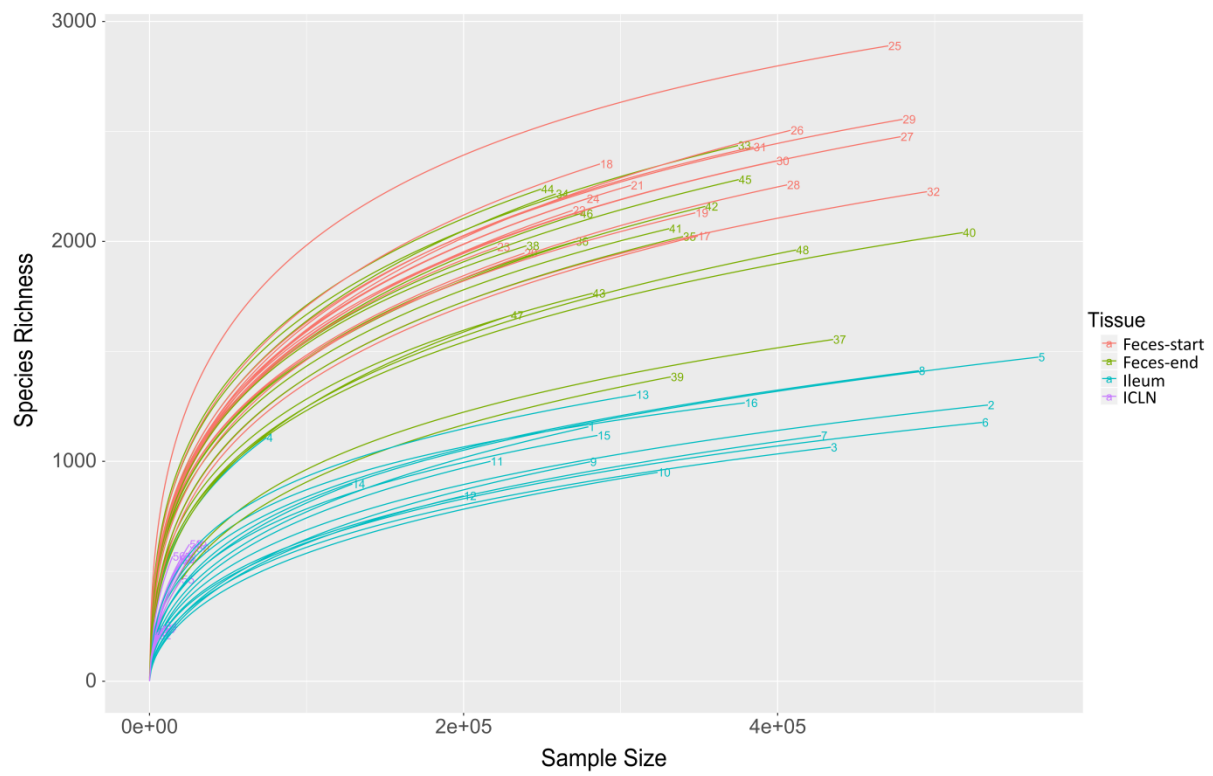

Supplementary Figure S1: Rarefaction curve of individual 16S rRNA gene libraries.

Supplement: Supplementary file 11 [file Image_1.pdf]
